# Comparison of Trunk and Lower Limb Muscle Activation Between Non-Motorized Treadmill and Flat Ground Walking at Varying Intensities in Patients with Stroke

**DOI:** 10.3390/bioengineering13070735

**Published:** 2026-06-25

**Authors:** Minkwon Cho, Taewoong Jeong, Yijung Chung

**Affiliations:** 1 Wirye Hospital, Seongnam 13647, Republic of Korea; 2 Department of Rehabilitation Medicine, Soonchunhyang University Bucheon Hospital, Bucheon 14584, Republic of Korea; 3 Department of Physical Therapy, College of Health and Welfare, Sahmyook University, Seoul 01795, Republic of Korea

**Keywords:** stroke, electromyography, non-motorized treadmill, walking intensity

## Abstract

Although considerable research has investigated non-motorized treadmills (NMTs), most studies have focused on healthy adults or athletes. This study aimed to compare trunk and lower limb muscle activation during walking on an NMT and flat ground (FG) at different exercise intensities in patients with stroke. Eighteen patients with stroke participated in this within-subject, repeated-measures experimental study conducted at a single hospital. Participants performed walking trials under six randomized conditions, comprising both NMT and FG walking at intensities of 20%, 40%, and 60% of heart rate reserve (HRR). Muscle activation of the affected-side erector spinae, internal oblique, gluteus medius, gluteus maximus, vastus medialis oblique, biceps femoris, and lateral gastrocnemius was assessed. Walking on the NMT resulted in significantly greater overall muscle activation than walking on FG (*p* < 0.05). In addition, significant differences in trunk and lower limb muscle activation were observed across HRR levels during both NMT and FG walking (*p* < 0.05), indicating that exercise intensity influenced neuromuscular responses. These findings suggest that NMT walking, particularly at higher intensities, acutely increases neuromuscular demands, providing preliminary evidence for its potential application as a demanding walking condition for stroke rehabilitation.

## 1. Introduction

Stroke is a central nervous system disorder caused by sudden bleeding or ischemia in brain vessels, resulting in inadequate blood supply to brain tissue and leading to partial loss of brain function and functional disabilities [1]. People affected by stroke often experience sensory, motor, and cognitive impairments, which make it difficult for them to perform daily activities [2]. Gait impairment is one of the most common symptoms of stroke, with approximately 60% of patients with stroke experiencing ongoing difficulties with walking [3]. Patients with stroke have difficulty shifting and supporting their body weight onto the affected side during walking due to muscle weakness and balance issues [4].

Muscle weakness in patients with stroke is a limiting factor for functional ability [5] and it is closely related to walking [6]. Measurement of muscle strength is therefore a crucial factor in predicting walking speed and endurance in patients with stroke [7]. The hip abductor provides stability to the hip joint during the stance phase of walking, and proper control of the hip abductor is crucial for balance recovery and independent walking [8]. The hip extensor shows increased muscle activation as walking speed increases [9], and it contributes significantly to stabilizing the knee joint and maintaining posture [10]. The strength of the knee extensor and flexor is closely related to walking speed [11], and during walking, simultaneous contraction of the knee extensor and flexor at the initial stance phase provides stability to the lower limb [12].

Treadmill training used in the rehabilitation of patients with stroke offers the advantage of allowing patients to perform appropriate aerobic exercise even in limited spaces. It allows for repetitive task-oriented training and provides an environment similar to flat ground (FG) walking, which contributes to improving balance and walking in patients with stroke [13]. Body-weight-supported treadmill training induces longer single-limb support on the affected side compared to FG walking training and improves plantar flexor spasticity, thereby effectively enhancing balance and walking quality [14]. Treadmill training at a comfortable speed shows muscle activation patterns similar to FG walking [15], while training at higher speeds requires higher muscle activation in the trunk and lower limbs [16]. Furthermore, treadmill training with increasing speed and incline has been shown to be effective in enhancing motor control in patients with stroke and healthy individuals [17]. Treadmills are widely used in clinical settings and in research for stroke rehabilitation utilizing motorized mechanisms.

A non-motorized treadmill (NMT) is a treadmill that operates without a motor and is powered by the user’s own force [18]. Previous studies have indicated that NMT training in healthy adults is effective in strengthening the quadriceps femoris compared to conventional treadmills [19]. Another study reported that walking on an NMT showed higher muscle activation in the lower limbs compared to walking on a conventional treadmill and FG [20]. An NMT operates without a motor and provides mechanical resistance, requiring effort from the user [21]. Given that it is powered by the user’s own force, NMT walking for patients with stroke necessitates the use of both the non-affected and affected lower limbs. Despite these advantages, there is a lack of research conducted on NMTs for patients with stroke.

Patients with stroke often lack normal and voluntary muscle contraction abilities, making it difficult to control appropriate timing and muscle activation intensity [22]. This is caused by impairments in the number of motor units, the recruitment of different types of contracting muscle fibers, and the rate of firing [23]. Montgomery et al. [20] compared the muscle activation around the knee and ankle during NMT walking with conventional treadmill walking and FG walking in healthy adults. However, they did not examine muscle activation in the trunk and hip, which play a crucial role in balance during walking. Therefore, it is necessary to examine the muscle activation in the lower limb and trunk during NMT walking in patients with stroke. In this study, the aim is to provide foundational data for gait rehabilitation in patients with stroke by comparing and analyzing the muscle activation in the trunk and lower limbs on the affected side during the stance phase of NMT and FG walking.

## 2. Materials and Methods

### 2.1. Participants

This study was conducted with 18 patients with stroke who met the selection criteria and were hospitalized at K Hospital in Gyeonggi Province. The selection criteria for the study participants included patients with stroke who were at least six months post-stroke, had a Brunnstrom stage of IV or higher, were able to walk independently for more than 10 min, and had a score of 21 or higher on the Korean version of the Mini-Mental State Examination (MMSE-K), indicating they could communicate and follow instructions [24]. Patients were excluded if they had walking difficulties due to orthopedic surgery or disabilities in either lower limb, ankle contractures, walking difficulties caused by conditions other than stroke, vestibular or cerebellar disorders, or visual or auditory impairments [25].

### 2.2. Study Procedure

Before conducting the experiment, the study conditions and procedures were thoroughly explained to the participants, and the experiment proceeded only after they fully understood and signed the consent form. This study conformed to the ethical guidelines of the 1975 Declaration of Helsinki and was approved by the Institutional Review Board of S University (Approval No. 2-1040781-AB-N-01-2016136HR). This study was registered with the Clinical Research Information Service, and adheres to the World Health Organization International Clinical Trials Registry Platform (WHO-ICTRP) guidelines (registration number: KCT0010318).

To determine the heart rate reserve (HRR) of the study participants, their resting heart rate was measured using a POLAR heart rate monitor (Polar RS400SD; Polar Electro, Kempele, Finland) after they had rested sufficiently for 1 h before the experiment. The HRR for each participant was calculated based on their resting heart rate. To normalize muscle activation, electrodes were attached to the affected side’s erector spinae (ES), internal oblique abdominis (IO), gluteus medius (GMed), gluteus maximus (Gmax), vastus medialis oblique (VMO), biceps femoris (BF), and lateral gastrocnemius (LG), and maximum voluntary isometric contraction (MVIC) was measured [26]. Foot switches were attached to the heel and the first metatarsal of the affected side. Participants walked on the NMT and on FG at 20%, 40%, and 60% of their HRR. After maintaining each walking condition for 30 s, the muscle activation during the stance phase of 10 walking cycles was measured and averaged. To minimize muscle fatigue due to the exercise, a 1 min rest was provided after each walking condition. The allowable range for heart rate was set to ±5 bpm. Walking at 20%, 40%, and 60% of HRR on the NMT and FG was performed in a random order using a random number table. To ensure participant safety and standardize experimental conditions, assistive device and handrail use was strictly uniformized across all trials. All 18 participants utilized a light-touch handrail or guide support during both NMT and FG walking to mitigate fall risks while minimizing structural body-weight unloading. None of the participants relied heavily on a cane or required structural physical assistance for weight bearing, as the inclusion criteria required the ability to walk independently for at least 10 m [27]. To monitor walking intensity, prevent falls, and ensure safety, one assistant stood beside each participant to supervise.

### 2.3. Non-Motorized Treadmill and Target Heart Rate

**Figure 1:**
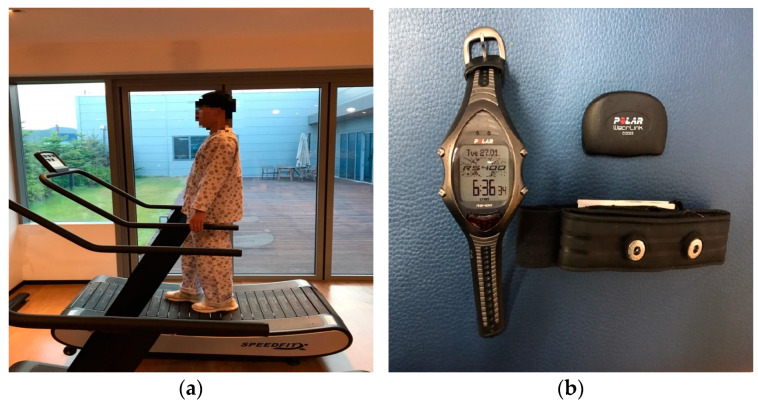
(**a**) Non-motorized treadmill; (**b**) POLAR heart rate monitor.

The NMT (Speedfit treadmill, SPT-1000C; Drax Inc., Anyang, Republic of Korea) is a treadmill with a curved surface, where the speed is determined by the user’s walking pace without the use of a motor or inverter (Figure 1a). The NMT features a curved surface that causes the incline angle to increase and speed to increase when moving forward, while speed decreases or stops when moving backward. The user can increase or decrease the speed according to their intention, and maintaining speed requires a consistent walking pattern. During both FG and NMT walking trials, the walking speed was not fixed but was continuously adjusted based on real-time heart rate monitoring. Participants wore a wireless heart rate monitor, and investigators provided verbal feedback to help them increase or decrease their walking pace to maintain their heart rate strictly within the targeted HRR zones for each condition.

In this study, heart rate monitors were attached to the participants’ chests to measure resting heart rate (Figure 1b). HRR was then calculated using the Karvonen method. Participants walked at 20%, 40%, and 60% of their HRR on both the NMT and FG, and muscle activation was measured for each muscle [28]. The target heart rate for each walking intensity was calculated using the Karvonen formula: Target Heart Rate = (Maximum Heart Rate − Resting Heart Rate) × (Intensity percentage/100) + Resting Heart Rate. The age-predicted maximum heart rate was estimated as Maximum Heart Rate = 220 − age in years. Regarding medical confounding factors, patients taking cardiovascular medications that directly alter heart rate responses, such as beta-blockers, were strictly excluded during the initial screening phase to ensure the validity of the heart rate reserve calculation.

### 2.4. Surface Electromyography

To measure muscle activation during the stance phase of walking, electromyography (EMG) was used with the Telemyo 2400 G2 Telemetry EMG system (Noraxon, Inc., Scottsdale, AZ, USA). Muscle activation of the affected side’s ES, IO, GMed, GMax, VMO, BF, and LG was assessed. The sampling rate for the EMG signals was set to 1500 Hz, with a frequency bandwidth of 20 to 500 Hz. In this study, the recorded EMG signals were processed using Myoresearch XP Master Edition software (Version 3.18, Noraxon, Inc., Scottsdale, AZ, USA), applying full wave rectification followed by the calculation of the root-mean-square values over a 300 ms window.

The electrode placement was as follows: for the ES, the electrodes were attached 3 cm lateral to the spinous process of the third lumbar vertebra on the affected side, aligned parallel to the muscle fibers. For the IO, the electrodes were positioned on the area just above the inguinal ligament, midway between the anterior superior iliac spine and the pubic symphysis. The electrodes for the GMed were attached 2 cm apart and aligned parallel to the muscle fibers at the proximal one-third between the iliac crest and the greater trochanter. The electrodes for the GMax were attached obliquely and parallel to the muscle fibers, 3 cm from the height of the greater trochanter at the midpoint between the greater trochanter and the sacrum. The electrodes for the BF were placed 2 cm apart, parallel to the muscle fibers, at the midpoint between the knee and the gluteal crease on the posterior thigh. The electrodes for the VMO were placed 4 cm superior and 3 cm medial from the superior 55-degree angle of the vertical line above the patella. The electrodes for the LG were attached 2 cm apart, parallel to the muscle fibers, at the lateral 2 cm of the posterior central region of the lower leg, below the knee [29]. For EMG normalization, the MVIC was measured using standardized manual muscle testing positions. To maximize reliability and minimize paretic compensation, participants performed three maximum trials for each muscle, with each contraction held for 5 s. A 60 s rest interval was provided between trials to prevent muscular fatigue. Standardized verbal encouragement was consistently provided by an experienced physical therapist. The highest root-mean-square (RMS) value over a 1 s stable window from the three trials was accepted as the baseline MVIC [26].

The normalization method involved measuring the EMG signals during 10 gait cycles at 30 s each, corresponding to 20%, 40%, and 60% of the HRR. The EMG signals extracted from the stance phase on the affected side were divided by the MVIC values to normalize them to %MVIC. Foot switches were used to confirm the stance phase during the gait cycles.

### 2.5. Statistics Analysis

Statistical analysis was performed using SPSS software (version 22.0). Data are expressed as the mean and standard deviation. Descriptive statistics were calculated for all variables. A repeated-measures analysis of variance (ANOVA) was conducted with two within-subject factors, walking method and walking intensity, to analyze trunk and lower-limb muscle activation. The Mauchly test of sphericity was monitored to verify the sphericity assumption; when the assumption was violated, Greenhouse–Geisser-corrected degrees of freedom were utilized. For main and interaction effects, the full statistical structure including degrees of freedom, *F*-statistic, *p*-value, and partial eta squared was reported. To adjust for multiple comparisons and minimize Type I errors, Bonferroni correction was applied for all post hoc pairwise comparisons. To justify the sample size, a post hoc power analysis was performed using G-Power software (version 3.1.9.7). For the repeated-measures design with 18 participants and 6 measurement conditions, assuming a strong effect size (partial eta squared greater than 0.14, Cohen f greater than 0.40) at a significance level of 0.05, the statistical power (1 − beta) exceeded 0.80 for the primary muscle activation outcomes, indicating that the sample size was statistically adequate to detect the main experimental effects. The significance level for all statistical tests was set at *p* < 0.05.

## 3. Results

The participants consisted of 18 individuals, including 11 men and seven women, with a mean age of 53.69 years, an average height of 165.5 cm, and an average weight of 65.17 kg. The stroke types included ischemic stroke (10 cases) and hemorrhagic stroke (eight cases). The average time since stroke onset was 21.39 months (Table 1).

Muscle activation values for all muscles are presented in Table A1. Among these, the ES and GMed did not show significant interaction effects between HRR levels and walking methods, nor did they show significant main effects of walking methods, as confirmed by the repeated-measures ANOVA results (Table A2). However, there was a significant main effect of HRR levels (*p* < 0.05). The muscle activation of the GMax, BF, and IO showed no significant interaction effect between HRR levels and walking methods, but both the main effects of HRR levels and walking methods were significantly different (*p* < 0.05). The muscle activation of the VMO showed no significant interaction effect between HRR levels and walking methods, but both the main effects of HRR levels and walking methods were statistically significant (*p* < 0.05). Muscle activation of the LG did not show significant interaction effects between HRR levels and walking methods, nor did it show a significant main effect of walking methods. However, there was a significant main effect of HRR levels (*p* < 0.05).

## 4. Discussion

This study was conducted using a within-subject, repeated-measures experimental design to compare trunk and lower-limb muscle activation on the affected side during the stance phase of walking on an NMT versus FG in patients with stroke, according to walking intensity. As a result, walking on an NMT showed higher muscle activation in the IO, GMax, VMO, and BF compared to walking on FG. Additionally, muscle activation in both the trunk and lower limbs increased with higher walking intensity.

Unlike traditional treadmills, an NMT lacks a motor and requires users to propel the belt and control the speed using their own effort [30]. Unlike walking and running on traditional treadmills or FG, walking on an NMT results in higher muscle activation in the lower limbs [20]. For both healthy adults and patients with stroke, comfortable walking speeds on an NMT are slower compared to those on FG [31]. Additionally, the distance covered in a 6 min walk test using an NMT is shorter compared to the distance covered in a 6 min walk test on a FG [21].

Montgomery et al. [20] compared walking, jogging, and running on FG, traditional treadmills, and NMT in healthy adults to investigate the muscle activation of the rectus femoris, semitendinosus, tibialis anterior, and soleus. As a result, walking on an NMT showed significantly higher muscle activation of the rectus femoris and semitendinosus compared to walking on FG and traditional treadmills. In this study, walking on an NMT showed higher muscle activation in the NMO and BF compared to walking on FG. The NMT, compared to FG, has a slight incline on the floor, which simulates climbing an incline [21]. Additionally, since the user must maintain forward movement by pressing their foot against the belt during the stance phase, this requires increased force on the floor, which places demands on the hip and knee joints [19,30]. The NMT has an incline of approximately 5 to 10 degrees [30], which is likely to have increased the muscle activation of the GMax, VMO, and BF.

The proprioceptive information from the NMT, due to its different belt material and the user-controlled belt movement, along with the fear of falling while using the NMT, contributed to a slower comfortable walking speed on the NMT compared to the comfortable walking speed on FG [31]. Balance exercises on unstable surfaces stimulate proprioceptive sensations and increase trunk muscle activation [32]. In this study, the muscle activation of the IO was found to be higher during walking on the NMT compared to walking on FG. This suggests that walking on an NMT requires individuals to actively control the movement of the belt to maintain balance [30]. Additionally, the dynamic support surface of the NMT likely increases the activation of the IO to enhance trunk stability compared to walking on a stable, FG.

Janaudis-Ferreira et al. [21] compared the distance and rating of perceived exertion (RPE) for leg fatigue during a 6 min walk test conducted on an NMT versus a conventional corridor. They found that the distance covered on the NMT was significantly reduced, while leg fatigue and RPE were significantly increased. This suggests that walking on an NMT is more intense compared to walking on FG. In previous research comparing walking at speeds of 1 mph, 1.5 mph, 2.5 mph, and 3.5 mph on an NMT and conventional treadmills, it was found that the NMT resulted in significantly higher heart rates, oxygen consumption, and RPEs, while the oxygen saturation of the gastrocnemius significantly decreased [33]. This indicates that walking on an NMT results in higher muscle fatigue and a greater exercise intensity compared to walking on a conventional treadmill. The high exercise intensity of NMT walking in this study is believed to have affected the muscle activation of both the trunk and lower extremities [34].

However, it is critical to distinguish whether these observed increases in muscle activation reflect improved neuromuscular recruitment or potentially inefficient movement strategies, such as abnormal co-contraction and compensatory efforts inherent in stroke gait patterns. While higher electromyographical signals indicate elevated acute neuromuscular demands during NMT walking, they do not automatically equate to long-term clinical or functional recovery. The elevated activation could partially represent a compensatory response to the unfamiliar mechanical constraints and balance demands of the curved treadmill surface. While we have attributed the observed increases in muscle activation to factors such as the curved belt incline, belt resistance, and inherent exercise intensity of the NMT, these interpretations remain speculative as our study design did not directly measure walking kinematics, ground reaction forces, or precise belt position. It is possible that unmeasured variables, including subtle variations in stride parameters or individual compensatory movement strategies, may have acted as mediators or confounders influencing our findings. Further research utilizing comprehensive biomechanical analysis is warranted to isolate these mechanical and physiological contributors.

In this study, walking at 60% of the HRR significantly increased muscle activation in the trunk and lower limbs compared to walking at 40% and 20%. Additionally, walking at 40% of the HRR increased muscle activation in the IO, GMax, GMed, VMO, and LG compared to walking at 20%. In patients with stroke, fast walking requires significant muscle activation of the quadriceps femoris to absorb the impact of body weight [35]. Additionally, the hip extensor, abductor, and hamstrings provide propulsion during the initial stance phase [36]. Previous research has reported that as walking speed increases, ground reaction forces rise, and the contributions of the GMax, GMed, hamstrings, vastus lateralis oblique, VMO, and gastrocnemius to weight support and forward propulsion are also elevated [37]. Another study reported that the increase in ground reaction forces during walking is associated with greater muscle activation in the hip abductor and ankle plantarflexor in patients with stroke [38]. In this study, walking intensity was determined based on heart rate, which reflects an increase in speed. The increase in speed was associated with higher muscle activation in both the trunk and lower limbs.

A limitation of this study is the relatively small sample size that was obtained from a single institution, which may limit the generalizability of the findings. Although the post hoc power analysis confirmed sufficient power (greater than 0.80) to detect large main effects of walking surface and intensity, the lack of significant interaction effects in certain muscles might be attributed to a lack of statistical power to detect subtler, differential responses. Future multi-center studies with larger sample sizes are required to verify potential interaction effects and extend generalizability. Additionally, given that this study utilized a within-subject, repeated-measures experimental design, future research should focus on investigating the long-term training effects of NMT interventions in patients with stroke.

## 5. Conclusions

In conclusion, NMT walking elicits significantly higher acute trunk and lower-limb muscle activation compared to FG walking in patients with stroke. These findings provide preliminary biomechanical evidence regarding immediate neuromuscular responses, rather than long-term training effects. Therefore, NMT walking could be suggested as a task-specific evaluation or a demanding walking condition that acutely increases muscular demands, although longitudinal studies are required to confirm its therapeutic effectiveness as a training intervention.

## Figures and Tables

**Table 1:** General and clinical characteristics of participants.

Variable	Number of Subjects/Value
Gender	
Male	11
Female	7
Age (years)	53.69 ± 10.59
Height (cm)	165.5 ± 8.42
Weight (kg)	65.17 ± 8.75
Stroke type	
Ischemia	10
Hemorrhage	8
Paralyzed area	
Right	9
Left	9
Onset (months)	21.39 ± 15.53
MMSE-K (points)	24.55 ± 2.64
MAS (points)	0.69 ± 0.82

Values are presented as number or mean ± standard deviation. MMSE-K, Korean version of Mini-Mental State Examination; MAS, Modified Ashworth Scale.

## Data Availability

The data that support the findings of this study are available from the corresponding author upon reasonable request.

## Appendix A

**Table A1:** Muscle activation according to target heart rate levels and walking methods.

Muscle		Muscle Activation (%MVIC)		
		60% HRR	40% HRR	20% HRR
ES	NMT	37.43 ± 14.04 ^b,c^	32.79 ± 13.97	28.8 ± 11.78
	FG	34.64 ± 10.74 ^b,c^	29.87 ± 9.65 ^b^	29.38 ± 9.50
GMed	NMT	33.36 ± 16.68 ^b,c^	27.65 ± 16.31 ^b^	23.44 ± 13.34
	FG	31.13 ± 14.35 ^b,c^	27.00 ± 13.98 ^b^	24.10 ± 11.71
GMax	NMT	28.70 ± 11.36 ^b,c^	25.07 ± 12.04 ^a,b^	21.80 ± 10.49 ^a^
	FG	21.99 ± 8.04 ^b,c^	17.84 ± 7.90 ^b^	16.20 ± 7.29
BF	NMT	33.69 ± 20.00 ^b,c^	27.46 ± 18.19 ^a^	23.00 ± 13.40
	FG	25.49 ± 10.17 ^b,c^	19.33 ± 7.98 ^b^	18.31 ± 6.45
IO	NMT	22.77 ± 11.26 ^b,c^	23.82 ± 9.44 ^a,b^	20.68 ± 8.68 ^a^
	FG	22.08 ± 8.97 ^b,c^	18.23 ± 8.05 ^b^	16.44 ± 6.68
VMO	NMT	39.43 ± 20.12 ^b,c^	32.08 ± 16.47 ^a,b^	26.14 ± 14.05 ^a^
	FG	30.45 ± 11.56 ^b,c^	25.77 ± 11.55 ^b^	22.05 ± 9.93
LG	NMT	46.21 ± 20.49 ^b,c^	42.81 ± 18.60 ^b^	37.24 ± 17.89
	FG	42.64 ± 17.64 ^b,c^	35.53 ± 13.91 ^b^	31.03 ± 11.32

Values are presented as mean ± standard deviation. MVIC, maximum voluntary isometric contraction; HRR, heart rate reserve; NMT, non-motorized treadmill; FG, flat ground; ES, erector spinae; GMed, gluteus medius; GMax, gluteus maximus; BF, biceps femoris; IO, internal oblique; VMO, vastus medialis oblique; LG, lateral gastrocnemius. ^a^ Significant difference compared with FG (*p* < 0.05). ^b^ Significant difference compared with 20% HRR (*p* < 0.05). ^c^ Significant difference compared with 40% HRR (*p* < 0.05).

**Table A2:** Two-way repeated measures ANOVA results and post hoc comparisons for muscle activation.

Muscle	Factor	df	*F*	*p*	η*p*^2^
ES	Intensity (%HRR)	(2, 34)	3.27	0.042	0.161
	Method (NMT vs. FG)	(1, 17)	0.57	0.451	0.033
	Interaction (%HRR × Method)	(2, 34)	0.26	0.774	0.015
GMed	Intensity (%HRR)	(2, 34)	3.10	0.049	0.154
	Method (NMT vs. FG)	(1, 17)	0.07	0.791	0.004
	Interaction (%HRR × Method)	(2, 34)	0.09	0.914	0.005
GMax	Intensity (%HRR)	(2, 34)	3.92	0.023	0.187
	Method (NMT vs. FG)	(1, 17)	12.22	0.001	0.418
	Interaction (%HRR × Method)	(2, 34)	0.07	0.933	0.004
BF	Intensity (%HRR)	(2, 34)	4.04	0.020	0.192
	Method (NMT vs. FG)	(1, 17)	7.11	0.009	0.295
	Interaction (%HRR × Method)	(2, 34)	0.19	0.891	0.011
IO	Intensity (%HRR)	(2, 34)	4.62	0.012	0.214
	Method (NMT vs. FG)	(1, 17)	9.02	0.003	0.347
	Interaction (%HRR × Method)	(2, 34)	0.074	0.929	0.004
VMO	Intensity (%HRR)	(2, 34)	5.15	0.007	0.232
	Method (NMT vs. FG)	(1, 17)	5.45	0.021	0.243
	Interaction (%HRR × Method)	(2, 34)	0.26	0.771	0.015
LG	Intensity (%HRR)	(2, 34)	3.33	0.040	0.164
	Method (NMT vs. FG)	(1, 17)	3.05	0.084	0.152
	Interaction (%HRR × Method)	(2, 34)	0.18	0.832	0.011

HRR, heart rate reserve; NMT, non-motorized treadmill; FG, flat ground; ES, erector spinae; GMed, gluteus medius; GMax, gluteus maximus; BF, biceps femoris; IO, internal oblique; VMO, vastus medialis oblique; LG, lateral gastrocnemius.
